# P-1687. Antibiotic Prophylaxis before Dental Procedures to Prevent Prosthetic Joint Infections

**DOI:** 10.1093/ofid/ofae631.1853

**Published:** 2025-01-29

**Authors:** Mariam Raheem, Ryan Kuhn, Suganthini Krishnan Natesan, Sorabh Dhar

**Affiliations:** Wayne State University Detroit Medical Center, Bloomfield, Michigan; John D Dingell VA Medical Center, Detroit, Michigan; John D Dingell VAMC/Wayne State University, Detroit, Michigan; Wayne State University/Detroit Medical Center, John Dingell VAMC, Detroit, Michigan

## Abstract

**Background:**

Antibiotic prophylaxis before invasive dental procedures (IDP) to reduce the risk of late prosthetic joint infections (PJI) is a controversial, yet common practice despite lack of supporting evidence. Antibiotics are no longer recommended, except for certain high-risk populations, highlighted in dental and orthopedic guidelines. To assist providers, the American Academy of Orthopedics (AAOS) has developed an algorithmic clinical decision support tool (CDS) “appropriate use criteria” matrix. However, uptake of these criteria for dental prophylaxis and antibiotic prescribing in routine dental care of patients with prosthetic joints remains unclear.

**Figure:**
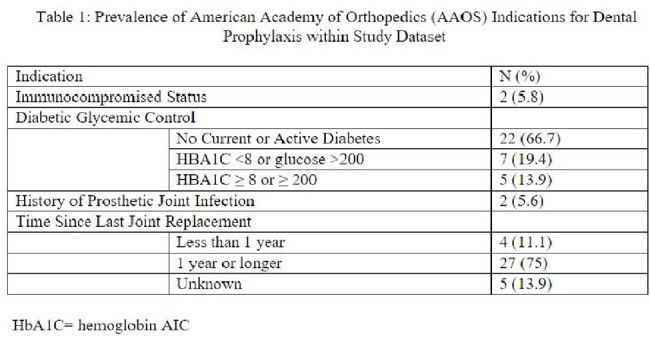

**Methods:**

This retrospective review at a Veterans Affairs hospital evaluated use of amoxicillin and clindamycin over 16 months for prophylaxis prior to high risk dental procedures in patients with prosthetic joints. Clinical data was reviewed for 5 indications defined by the AAOS including: 1) Invasive dental procedure (manipulation of the gingiva or periapical tissues), 2) Immunocompromised status, 3) Diabetic glycemic control (as defined by hemoglobin A1C ≥ 8 or blood glucose ≥ 200), 4)previous history of (PJI), and 5)timing of the joint replacement (≥ 1 year). The use of the antibiotic was categorized into 3 groups per AAOS guidance: 1). Appropriate, 2). Maybe Appropriate, 3). Rarely appropriate. Records were also reviewed for subsequent joint infection, antibiotic adverse events, and development of Clostridioides difficile infection (CDI).

**Results:**

Indications for dental prophylaxis among the 36 cases reviewed are shown in table 1. Of the records reviewed, 0 (0%) were deemed “appropriate”, 2 (5.6%) “maybe appropriate”, and 34 (94.4%) “rarely appropriate.” No joint infections, adverse antibiotic events, CDI were noted in the subsequent 90 days.

**Conclusion:**

Despite the availability of a clinical decision support tool for prescribing prophylactic antibiotics to patients with prosthetic joints, 95% of antibiotics were found to be unnecessary per current AAOS guidelines. Provider feedback and updated evidence-based recommendations are needed to promote appropriate antibiotic use.

**Disclosures:**

**All Authors**: No reported disclosures

